# Role of Percutaneous Nephrostomy in Advanced Cervical Carcinoma with Obstructive Uropathy: A Case Series

**DOI:** 10.4103/0973-1075.53510

**Published:** 2009

**Authors:** Kamlesh Mishra, Ava Desai, Shilpa Patel, Meeta Mankad, Kalpana Dave

**Affiliations:** Department of Gynecology Oncology, Gujarat Cancer and Research Institute, Ahmadabad, India

**Keywords:** Cervical cancer, Obstructive uropathy, Percutaneous nephrostomy

## Abstract

**Aims and Objective:**

Over 70% of the cases present in advanced stages of the disease and are associated with poor prognosis and high mortality rates. In many of them, it is difficult to offer definitive treatment as they present in uremia due to associated obstructive uropathy. There are no clear-cut guidelines for performing percutaneous nephrostomy (PCN) in patients of advanced cervical cancer. The results are unpredictable in terms of benefits achieved in these cases. Thus, we evaluated our experiences with PCN in the management of cervical cancer patients presenting with obstructive uropathy.

**Material and Methods:**

15 patients of cervical cancer with obstructive uropathy and deranged renal functions were retrospectively evaluated for the role of PCN in their management

**Results:**

PCN was done in 15 patients of advanced cervical cancer. The mean age of patients was 44.5 years. Twelve (80%) patients presented primarily with advanced cervical carcinoma and obstructive uropathy. Three (20%) were already treated. Symptomatic improvement and significant fall of mean serum creatinine value from 7.5 mg% to 0.9 mg% over a period of 1-3 weeks was noted post PCN. Out of 12 patient with primary untreated advanced disease, curative treatment was possible in 3, palliative radiotherapy/chemo-therapy in 7 and only symptomatic treatment in 2 cases, after obstructive uropathy was managed with PCN insertion. Out of 3 already treated patients, 2 were disease free after curative radiotherapy/surgery. PCN was done to prevent permanent kidney damage in them. One patient was defaulter of curative radiotherapy. She had progressive residual disease. Complications like hemorrhage (20%), infection (26%), reinsertion for dislodgment/misplacement (53%), percutaneous leak or perinephric leak (20%), blockage of PCN (33%) were noticed.

**Conclusion:**

In spite of inherent, albeit manageable complications, PCN is a simple and safe technique. One of the major benefits observed was ability to administer either curative/palliative radiotherapy or chemotherapy in 85% of patients (11 out of 13 with disease). There was no active disease in remaining 2 patients. Therefore, the decision to attempt PCN in carefully selected cervical cancer patients is justified.

## INTRODUCTION

Cervical cancer is the most common female cancer in the developing countries and its incidence in India is about 32 per 100,000 women.[1] Over 70% of the cases present in advanced stages of the disease with associated poor prognosis and high mortality rates.[2] In many of them, it is difficult to offer definitive treatment as they present in uremia due to associated obstructive uropathy. This is due to either external compression or malignant involvement of lower ureters. These patients may have large primary advanced/recurrent/post treatment progressive residual disease. Obstructive uropathy was also sometimes observed in previously treated patients who had no evidence of recurrent disease, but developed hydronephrosis due to entrapment of ureters in pelvic fibrosis. Patients may be symptomatic or asymptomatic with high blood urea nitrogen (BUN), serum creatinine, and electrolytes. Urinary diversion by percutaneous nephrostomy (PCN) is the commonly practiced method, not only to improve renal function,[3] but also to improve quality of life and enable the patient to accept tumor specific palliative treatment in most and curative treatment in some. There are no clear-cut guidelines for PCN in patients of advanced cervical cancer. The results are unpredictable in terms of recovery of renal functions and benefit attained to administer subsequent radiotherapy or surgery or chemotherapy. Therefore, we evaluated our experience with PCN in the management of cervical cancer patients presenting with obstructive uropathy.

## MATERIALS AND METHODS

We retrospectively evaluated 15 patients of cervical cancer with obstructive uropathy and deranged renal functions. They underwent urinary diversion by PCN at Gujarat Cancer and Research Institute, Ahmedabad. In all cases the prognosis, subsequent treatment possibilities, and expected results were explained in detail. The need for PCN insertion was judged by ultrasound findings of hydronephrosis with obstruction of pelvic ureters, associated with high serum creatinine and BUN levels. Bilateral PCN was done by the urologist under ultrasound guidance. Until the improvement of the renal functions, the total fluid intake was restricted to 500 cc over output. Urine analysis and cultures from both PCN sites were performed at regular intervals. Positions of catheters were confirmed with X-ray KUB after the procedures. Serum creatinine and BUN were measured on Day 1, 7, and 14. In case of non-functioning PCN, a nephrostomogram was done to confirm the position of the catheter in the renal pelvis.

## RESULTS

Mean age of patients was 44.5 years (30-65 years). Patients were divided into Group A (Post-treatment) and Group B (untreated).

Group A [Table 1] had 3 patients, who had previously received definitive treatment (surgery/radio-therapy). One had undergone radical hysterectomy followed by adjuvant radiotherapy. She developed bilateral hydronephrosis 4 months after completion of therapy. The second patient received curative radiotherapy and developed right sided unilateral gross hydronephrosis. Both the patients did not show any evidence of recurrent disease. They were treated with urinary diversions with PCN to avoid permanent renal damage. In the first patient, retrograde trans-urethral ureteric D-J stenting was achieved after 14 days and the PCN catheters could be removed. In contrast, in the second patient (who received curative radiotherapy); D-J stenting was not possible. At present, she is continuing with PCN and later on permanent surgical diversion is planned. The third patient had residual disease after irregular, incomplete radiation for Stage IIIB. She reported with uremia and large residual disease involving the lower ureters. Bilateral PCN was done to relieve hydronephrosis followed by palliative chemotherapy.

**Table 1:** Description of patients developing obstructive uropathy after treatment of cervical cancer (Group A)

Type of treatment received and initial disease status	Number of patients	Duration between completion of treatment and obstructive uropathy (in months)	Disease status and further treatment
Curative radiotherapy Stage IIIb	1	4	No residual disease
Radical surgery followed by adjuvant radiotherapy Stage Ib2	1	4	No residual disease
Irregular, incomplete curative radiotherapy Stage IIIB	1	4	Progressive residual disease, on palliative chemotherapy

Twelve patients in Group B [Table 2] primarily presented with untreated advanced cancer cervix with obstructive uropathy and deranged renal function tests. All underwent PCN. Curative radiotherapy was given to 3 patients, palliative radiotherapy to 5 patients and palliative chemotherapy to 2 patients after PCN. In the remaining two patients of this group, only symptomatic treatment was given as disease was Stage IV and performance status was poor.

**Table 2:** Description of untreated patients, presented primarily with advanced disease with obstructive uropathy (Group B)

Treatment received after percutaneous nephrostomy	No. of patients	Specific treatment administered
Curative treatment	3	Curative radiotherapy
Palliative treatment	5	Palliative radiotherapy
	2	Palliative chemotherapy
Only symptomatic treatment	2	No specific treatment

Curative treatment was possible in form of radiotherapy and/or chemotherapy in 3 patients out of 12 (Group B) Stage IIIB cancer cervix patients. First had fast growing tumor, histopathologically small cell carcinoma of neuroendocrine type. She had peritoneal metastases and enlarged pelvic lymph nodes. Thus required aggressive treatment with multidisciplinary approach to give her a fair chance of survival. While being worked-up, she developed a fresh large metastasis peritoneal mass in lumbar region, acute renal failure with bilateral hydronephrosis. PCN was done. She completed radiotherapy (pelvic and cranial) and chemotherapy (BEP 6 cycles). She is disease free for 1 year after completion of treatment. Remaining 2 patients were Stage III B squamous cell carcinoma. After PCN, both received curative radiotherapy. Both are disease free for 4 and 7 months after completion of therapy, respectively. Total of 7/12 untreated patients received palliative treatment (radiotherapy to 5 and chemotherapy to 2) after PCN. In 2 patients of this group only symptomatic treatment was given as disease was Stage IV and performance status was poor.

There was bilateral ureteric obstruction in 14 patients (93%) and unilateral block in 1 (7%). PCN procedure was feasible in all cases. Besides the symptomatic improvement, the significant fall of mean serum creatinine value from 7.5mg% (2.7-12.5mg%) to 0.9 mg% (0.7-2.5 mg%) and of mean blood urea nitrogen from 41.2 mg% (22-65 mg%) to 14.36 mg%(11.5-28 mg%) over a period of 1-3 weeks was noted after PCN [Table 3]. After performing PCN, we normally admit the patient for a few days for observation. However, average duration of hospital stay in the present study was 31 days (10-102 days) as this included management of infection, re-insertions, diagnosis and assessment of disease status, and subsequent palliative or curative therapy.

**Table 3:** Description of renal functions parameters at various time periods

Renal functions	Day-o (mg/100ml)	Day-7 (mg/100ml)	Day-14 (mg/100ml)tt
Serum creatinine	7.5 (2.7-12.5)	1.2 (0.9-2.8)	0.9 m (0.7-2.5)
Blood urea nitrogen	41.2 (22-65)	16.25 (12-26)	14.36 (11.5-28)

One or more complications were noticed in 12 out of 15 patients (80%) cases. Three (20%) patients had no urine output unilaterally. In 8 (53%) cases, reinsertion was required. Infection was seen in 4 (26%) and was appropriately treated with antibiotics according to culture sensitivity reports. Perinephric space (3) and/or percutaneous leak (3) of urine occurred in 6 (40%). Mild to moderate hemorrhagic urine was noted in 3 (20%) patients. In 2 (13%) patients frank pus drained from unilateral catheters. Most patients required repeated flushing of catheters to maintain patency and stringent antiseptic dressings of local sites to prevent infections.

In group A (previously treated patients), 2 out of 3 (66%) were disease free. PCN was performed to avoid permanent renal damage in these potentially cured patients. In group B (untreated group), 7 out of 12 (58%) patients received palliative radiation/chemotherapy. Curative treatment was given in 3 out of 12 (25%) patients, with subsequent removal of PCN catheters, after tumor regression resulted in relief of ureteric obstruction.

## DISCUSSION

PCN was first described by Goodwin *et al.* in 1955.[4] The most important factors determining extent of recovery of renal function are extent and duration of obstruction.[5,6] In humans, partial recovery, stopping dialysis has been reported even after 7 months of complete obstruction.[7] Michael Hopkins[8] reported hydronephrosis to have prognostic value in Stage IIIB cancer cervix patients. In his study, 5 year survival rate in patients with normal IVP with no obstruction was 47%, with ureteric obstruction without renal failure was 29%. Contrary to this, all patients with ureteric obstruction with renal failure died within 16 months.

Obstructive uropathy with uremia with the risk of impending irreversible renal damage is a common presentation in a significant proportion of cervical cancer patients in developing countries. Percutaneous nephrostomy is useful especially in this situation, since retrograte ureteric stenting is often not possible. In the present study, PCN was feasible in all the 15 patients (100%). Very low failure rates of 0-3% have been reported in other studies too.[10,11] This indicates that though it is an invasive procedure, it is very simple and feasible. Its only contraindication is bleeding diathesis. We opted for bilateral PCN insertion over unilateral PCN or intraureteric catheterization as an emergency temporary method for renal function correction. In only one post radiotherapy patient with unilateral hydronephrosis, one- sided PCN was done. Hyppolite Jean-Claude[9] in his study of obstructive uropathy in gynecological malignancies found bilateral nephrostomy to be superior to unilateral nephrostomy and even to intraureteric stenting. So much so that, they suggested avoidance of intraureteric catheter placement in cervical cancer patients, as it was associated with 86% incidence of urosepsis, leading to death in 43%. In the present study with PCN, manageable complications were noted in 80% cases, almost same as reported in other studies 62-83%.[7,10] Literature reports cited 29-60% incidence of reinsertion.[11,12] It was noted in 53% cases in present study.

One of the most important advantages of PCN insertion noted here was that we could administer tumor specific treatment in 83%; i.e., curative radiotherapy in 3 and palliative radiotherapy/chemotherapy in 7 out of 12 untreated patients. Similar benefit although to lesser number of patients is noted in other study too, with 32% of the patients surviving long enough to undergo some form of treatment focused on the primary tumor after PCN.[13] In that study, PCN was found to be more useful in cervical cancer patients than those of bladder/prostrate cancers with obstructive uropathy.[13]

Most (93%) patients were of advanced stage cancer, compared to 60% patients of advanced (≥Stage IIB) stages.[11] Jonathan *et al*.,[11] in their study on PCN in gynecological malignancies had 31% patients in early stage of malignancy. The higher number of early staged patients with hydronephrosis could be due to inclusion of other gynecological malignancies too, besides cervical cancers. In our study, only one patient was of Stage IB1 and that too she developed hydronephrosis due to post treatment fibrosis.

Decision to do invasive PCN should be individualized in cervical cancer patients presenting with deranged renal functions as a result of obstructive uropathy, on the basis of availability of subsequent definitive treatment options. In previously treated patients with no recurrence, role of PCN as an emergency temporary measure to avoid renal failure is unquestionable. In carefully selected patients who present primarily with advanced disease it improves quality of life and prolongs survival by enabling tumor specific treatment. It increases acceptability to palliative radiotherapy or chemotherapy by correcting deranged renal functions. In small but definite number of cases, even curative treatment with long survival could be achieved. However, its role in recurrent or residual disease (where no further tumor directed treatment is available) seems controversial. The reasoning for avoiding PCN in such cases is that, it may be better to let the patient die peacefully due to uremia, rather than to prolong her suffering and agony with future fistulae and neuropathic pains, when we have no other treatment to offer.

Thus, decision-making process is complex. Counseling is essential and the wishes of the patient and her family have to be considered. Important factors to be taken into consideration before PCN decision, may be extent of disease and its status in terms of primary or recurrent or residual disease, availability of treatment options, patients performance status and associated medical co-morbid conditions. At the same time, it is always essential to explain in detail every possibility and unpredictability of results to patients and relatives.

Most studies reported in literature are retrospective, small sample based and non randomized. Therefore, the role of PCN in management of obstructive uropathy in cervical cancers actually needs to be defined more accurately in terms of survival benefit or quality of life improvement in large sample based, randomized, prospective trials.

## CONCLUSION

In treated and cured patients with long life expectancy, PCN was effective as a temporary means to save renal functions until retrograde stenting/surgical diversion could be offered. In treating naive patients, PCN was effective to improve renal function and allowed definitive treatment in many cases. In many cases, PCN could be removed after obstruction was relieved by tumor regression. In patients who have recurrence after completing definitive treatment and who presented with uremia, the only benefit of PCN is to prolong life. As no other definitive treatment could be offered even after PCN, the role of PCN in such cases is controversial. Hence, PCN is safe and feasible and should be done in carefully selected cases. It should be avoided in cases where it only serves to prolong suffering. Ultimately, the wish of patient needs to be respected.
